# Machine learning models can predict subsequent publication of North American Spine Society (NASS) annual general meeting abstracts

**DOI:** 10.1371/journal.pone.0289931

**Published:** 2023-08-22

**Authors:** Aazad Abbas, Olumide Olotu, Akeshdeep Bhatia, Denis Selimovic, Alireza Tajik, Jeremie Larouche, Henry Ahn, Albert Yee, Stephen Lewis, Joel Finkelstein, Jay Toor

**Affiliations:** 1 Division of Orthopaedic Surgery, University of Toronto, Toronto, ON, Canada; 2 Division of Orthopaedic Surgery, University of Western Ontario, London, ON, Canada; 3 School of Medicine, St. George’s University, University Centre, Grenada, West Indies; 4 Division of Orthopaedic Surgery, Sunnybrook Health Science Centre, Toronto, ON, Canada; 5 Division of Orthopaedic Surgery, St. Michael’s Hospital, Toronto, ON, Canada; 6 Division of Orthopaedic Surgery Toronto Western Hospital, Toronto, ON, Canada; 7 Department of Orthopaedic Surgery, University of Manitoba, Winnipeg, MB, Canada; Houston Methodist Academic Institute, UNITED STATES

## Abstract

**Background context:**

Academic meetings serve as an opportunity to present and discuss novel ideas. Previous studies have identified factors predictive of publication without generating predictive models. Machine learning (ML) presents a novel tool capable of generating these models. As such, the objective of this study was to use ML models to predict subsequent publication of abstracts presented at a major surgical conference.

**Study design/setting:**

Database study.

**Methods:**

All abstracts from the North American Spine Society (NASS) annual general meetings (AGM) from 2013–2015 were reviewed. The following information was extracted: number of authors, institution, location, conference category, subject category, study type, data collection methodology, human subject research, and FDA approval. Abstracts were then searched on the PubMed, Google Scholar, and Scopus databases for publication. ML models were trained to predict whether the abstract would be published or not. Quality of models was determined by using the area under the receiver operator curve (AUC). The top ten most important factors were extracted from the most successful model during testing.

**Results:**

A total of 1119 abstracts were presented, with 553 (49%) abstracts published. During training, the model with the highest AUC and accuracy metrics was the partial least squares (AUC of 0.77±0.05, accuracy of 75.5%±4.7%). During testing, the model with the highest AUC and accuracy was the random forest (AUC of 0.69, accuracy of 67%). The top ten features for the random forest model were (descending order): number of authors, year, conference category, subject category, human subjects research, continent, and data collection methodology.

**Conclusions:**

This was the first study attempting to use ML to predict the publication of complete articles after abstract presentation at a major academic conference. Future studies should incorporate deep learning frameworks, cognitive/results-based variables and aim to apply this methodology to larger conferences across other fields of medicine to improve the quality of works presented.

## 1 Introduction

In the scientific community, academic meetings serve as a means of disseminating new research information in a timely manner. In fact, 50–60% of the chapters of major orthopaedic textbooks included results from abstracts presented at international meetings [1]. Despite this fact, peer-reviewed journal articles remain the gold-standard for obtaining new research information [2]. As such, manuscript publication in a peer-reviewed journal is generally the goal for research presented at academic meetings and conferences.

The goal of abstracts eventually being published in peer-reviewed journals has led to a plethora of research dedicated to evaluating conference abstracts, with a major focus on evaluating publication rates of complete articles after presentation at an academic meeting [3–6]. An example is the 5-year abstract-to-publication ratio that was proposed as a metric to assess the scientific value and quality of academic meetings [7], with few studies addressing factors predictive of manuscript publication after abstract presentation [1,8]. As of now there have been no attempts to predict the chance of publication.

However, machine learning models (MLMs) have recently come to the fore in clinical research because of their predictive abilities, typically being used to predict clinical outcomes, improve system efficiencies and risk stratify patients [9–11]. As such, research on publication rates of conference abstracts is a novel opportunity to test MLMs in this emerging domain of academic medicine. Using MLMs, we can improve the quality of research by not only identifying factors predictive of publication as done with standard statistics, but further expand to determine the probability of publication. To date, MLMs have not been used to predict conference abstract publication after presentation at scientific meetings.

The North American Spine Society (NASS) Annual General Meeting (AGM) is an internationally recognized conference on spine surgery. As such, it provides an excellent platform for researchers and clinicians worldwide to showcase the latest advancements in spine surgery. This makes it an ideal conference to use to test the hypothesis of whether artificial intelligence (AI) methods such as MLMs may be used to detect subsequent publication after presentation.

As such, this study is primarily a feasibility study to evaluate the ability of MLMs to predict subsequent publication of abstracts presented at the NASS Annual General Meeting (AGM). Secondary objectives included stratifying the publication rates of these abstracts, as well as determining the predictive factors used by the most accurate model. We hypothesize that MLMs can be used to predict the publication rates of NASS AGM abstracts with acceptable accuracy.

## 2 Methods

### 2. 1 Selection of conference abstracts

All abstracts from the NASS AGM from 2013–2015 were reviewed. The 2015 annual general meeting was chosen as the upper limit as previous literature has shown that for most abstracts presented at conferences, subsequent publication most commonly occurs within 5 years of conference date [7]. Only abstracts directed at scientific research were included (i.e., workshops and educational exhibits were not included).

### 2. 2 Data extraction

The following information was extracted from the abstracts: abstract identification number, title, authors, number of authors, institution of first author, location (city, state/province, country), conference category, subject category, study type, data collection methodology, human subject research, and FDA approved indication.

### 2. 3 Identification of full-text publication

Abstracts were searched on the PubMed, Google Scholar, and Scopus databases for publication. Search date was inclusive of the deadline to submit an abstract to NASS AGM 2013 until 31st December 2020, which includes up to 1 year before NASS AGM 2013. The abstract title was searched and matched with authors. Only English language abstracts which were published in English or available in English were included.

Abstracts were searched with the first author’s last name and first initial to identify articles published with a similar title. If a manuscript was not discovered, the process was repeated for the second author. This would continue until all authors were searched. Abstracts were considered published if the following minimal criteria were met: 1) at least one author from the abstract was included in the manuscript; 2) at least one conclusion from the abstract was identical to the publication. An abstract was considered not published if the above method did not yield a manuscript. In cases where authors published multiple works with similar conclusions, all conclusions from the abstract would need to match the published paper.

In cases where a given author would have published multiple abstracts on the same topic, the methodology and conclusion were compared in detail to find an identical match to the original abstract. In cases where abstracts were presented at multiple categories of a conference, only the first presentation of the abstract was retained.

Subsequent publication was considered a binary outcome (i.e., yes if the abstract is published, no if not). For published abstracts, the following information was extracted: journal of publication, impact factor, date of publication, country of journal, NASS journal or not, and manuscript conclusion same as abstract. Impact factor was retrieved using the Web of Science Master Journal List (https://mjl.clarivate.com/home). If the impact factor was not found in the master list, the latest impact factor from the journal website or Google search was obtained and used. If the journal was no longer active, then the latest available information for it was used. The latest 2-year impact factor available (2018) was included.

Data extraction was performed using the Excel Software Package (Microsoft Corporation, Redmond, Washington). Continuous variables were collated and reported with a mean and standard deviation, while categorical variables were reported with total counts (N) and percentages.

### 2. 4 Prediction of publication

MLMs were trained to predict whether the abstract would be published (yes prediction) or not published (no prediction). The dataset was randomized and split into a training/testing ratio of 80/20 using stratified sampling to reduce class imbalance, with MLMs trained on the training set and tested on the testing set.

#### 2. 4. 1 Independent variables

Independent variables (features) selected for analysis include number of authors, institution of authors, location, conference category, subject category, study type, data collection methodology, and human subjects research. Refer to Table 1 for a detailed description of these variables. The correlation between features was examined by determining the Pearson correlation coefficient between each feature, with results visualized through a network plot. Features with absolute correlation coefficients > 0.5 were eliminated to avoid overfitting.

**Table 1:** Variables used as inputs (i.e., features) into the machine learning models.

Variable Name	Variable Type	Example Values
Conference category*	Categorical	Trauma, Deformity, Imaging, etc
Subject category	Categorical	Trauma, paediatrics, cancer, degenerative, inflammatory, minimally invasive, basic science, implants, others
Study Type	Categorical	Randomized controlled trial, cohort, cross-sectional, case control, case-report, clinical review, systematic review, scoping/literature review, basic science study, other
Data collection methodology	Categorical	Prospective, retrospective, other
Number of authors	Numerical	1, 2, 3,. . .
Country^†^	Categorical	USA, Canada, etc
Human subject research	Binary	Yes, No
FDA approved indication	Binary	Yes, No

*Details of categories omitted due to number of categories. Please refer to NASS AGM 2013–2015 abstracts for list of all categories.

^†^Countries were approximated by continents due to lack of data for each country.

ML: Machine learning.

#### 2. 4. 2 Missing data

For each predictor variable, an imputer was trained on the training set to input the missing data. A k-nearest neighbour algorithm was used to replace the missing data, with the weighted mean of the data used from the 10 nearest neighbours. Once trained and applied to the training set, the imputer was applied to the testing set to fill in any missing feature data.

#### 2. 4. 3 Models utilized

The MLMs that were used from the Caret package (Version 6.0.86) in R programming language were random forest, naive bayes, k-nearest neighbour, partial least squares, general boosted linear models, and neural networks [12–17].

#### 2. 4. 4 Model training and validation

Repeated k-fold cross validation was used on the training set to hyperparameter tune the models. Specifically, 10-fold cross validation with 5 repeats was used with a grid-search optimization approach to determine the model parameters such that the Receiver Operator Curve (ROC) metrics was maximized. Mean and standard deviation (SD) across of the following metrics were determined for each model across the repeated k-folds for the training dataset: area under the receiver operator curve (AUC), accuracy, sensitivity, specificity, positive predictive value (PPV) and negative predictive value (NPV).

#### 2. 4. 5 Model testing

Once trained and tuned, each model was applied to the test dataset. AUC, accuracy, sensitivity, specificity, PPV and NPV for each model was determined. In addition, confusion matrices representing the full distributions of the predictions for each model were determined. The model with the largest AUC was considered the most optimal. Statistical significance of these models during training and testing were determined by comparing the AUC metrics using analysis of variance (ANOVA) with Tukey’s test post-hoc sampling. Significance level was set at *p* < .05. Since there is no current literature defining the acceptable accuracy for MLMs in the context of publication rates, minimally acceptable definition for accuracy was set at 60% as this is slightly higher than a random prediction of 50%.

#### 2. 4. 6 Feature importance

Feature importance was extracted and normalized with respect to the most important feature (%) for the model with the highest AUC on the test set. All statistical analysis was completed using RStudio (RStudio Inc; Version 1.2.5042) [18], running R software (R Foundation, Version 3.6.3) [19].

## 3 Results

A total of 1119 abstracts were presented at NASS AGM between 2013–2015, with 553 (49%) of the abstracts published in scientific journals. The training and testing sets consisted of 896 and 223 abstracts, respectively. The median time to publication (years) was 1.25, with the mean time to publication being 1.33 (SD 1.49). The median impact factor of journals was 2.90, with the mean impact factor being 3.34 (SD 4.36). Across published abstracts, the median number of authors was 6, with the mean number of authors being 6.67 (SD 4.26). A breakdown of abstracts according to country, subject category, study type, data collection methodology, human subjects research and FDA approval status is found in Table 2. S1 Table and 2 provide these data for the training and testing set. There were no missing features in the dataset as shown by the aforementioned tables.

**Table 2:** Demographic breakdown of presented and published abstracts across the NASS AGM 2013–2015 for the entire dataset.

Variable	Presented (N)	Published (N)	Published (%)	Variable	Presented (N)	Published (N)	Published (%)
Country of publication				Study type			
USA	862	434	50.35	Other	298	144	48.32
Canada	58	30	51.72	Animal study	28	15	53.57
China	50	25	50.00	Basic science study	73	39	53.42
South Korea	24	13	54.17	Case-control	40	21	52.50
UK	20	8	40.00	Case-report/ series	214	103	48.13
Japan	17	9	52.94	Clinical review	62	24	38.71
Others	88	34	38.64	Cohort	245	130	53.06
Subject category				Cross-sectional	34	15	44.12
Other	478	254	53.14	Randomized controlled trial	88	43	48.86
MIS	144	65	45.14	Systematic review	37	21	56.76
Degenerative	131	68	51.91	Data collection methodology			
Implant	121	52	42.98	Retrospective	471	228	48.40
Basic science	108	50	46.30	Prospective	425	203	47.76
Paediatrics	53	24	45.28	Other	223	121	54.26
Trauma	50	26	52.00	FDA approved indication			
Human subjects research				Yes	94	44	46.80
Yes	547	249	45.52	No	69	31	44.93
No	572	303	52.97	n/a	956	477	49.90

AGM: Annual general meeting, FDA: Food and drug administration, MIS: Minimally invasive surgery, NASS: North American Spine Society, UK: United Kingdom, USA: United States of America.

### 3. 1 Publication results

The United States of America (USA) had the highest number of abstracts published (434, 50.4%). In terms of categories, ‘Other’ had the highest percentage of abstracts published (478, 53.1%), followed by ‘Trauma’ (50, 5%) and ‘Degenerative’ (131, 51.9%). Systematic review studies had the highest percentage of publication (37, 56.8%), followed by animal studies (28, 53.6%) and basic science studies (73, 53.4%). Studies with more than 10 authors had the highest percentage of publication (53.4%), followed by 6–10 authors (52.2%) and then less than 5 authors (46.1%).

### 3. 2 Machine learning predictions

**Fig 1:**
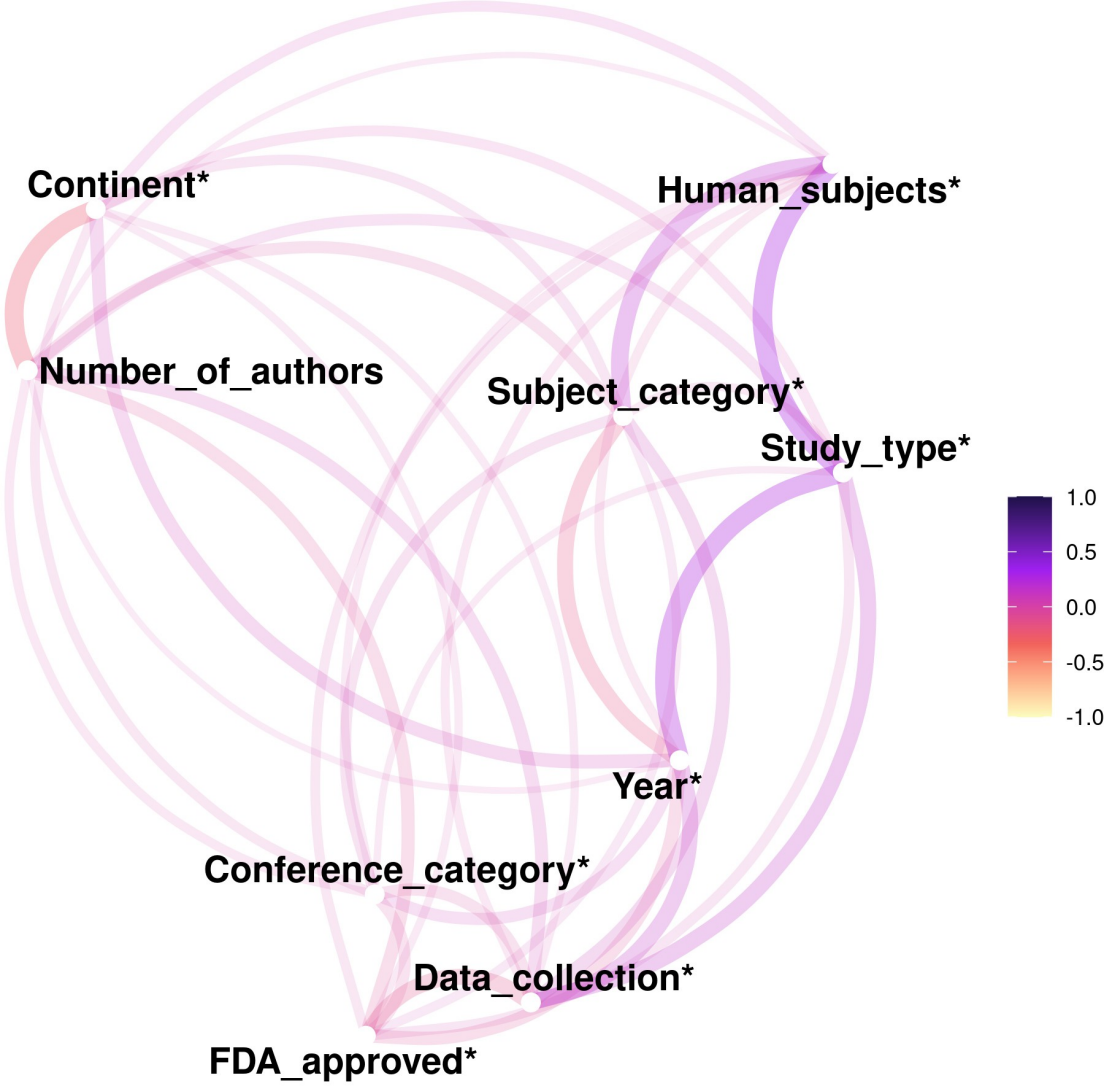
Network plot representing the correlation of features in the training set. Colour represents direction according to the scale on the right-hand side. Line thickness and proximity of features represent the strength of correlation. *Represent categorical features.

**Fig 2:**
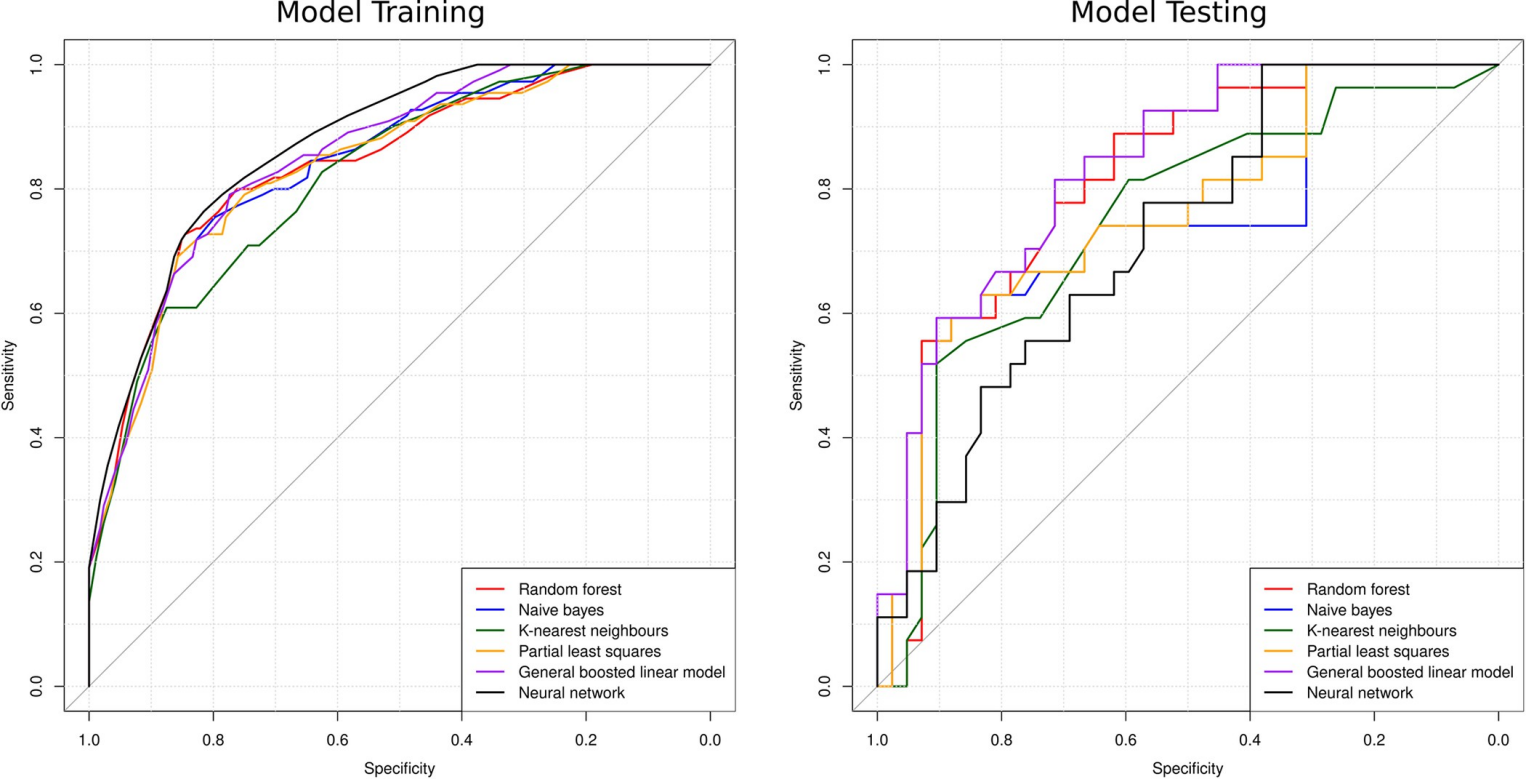
Receiver operator curve (ROC) plot for the models during training and testing. Models with larger areas under the ROC represent better models.

**Fig 3:**
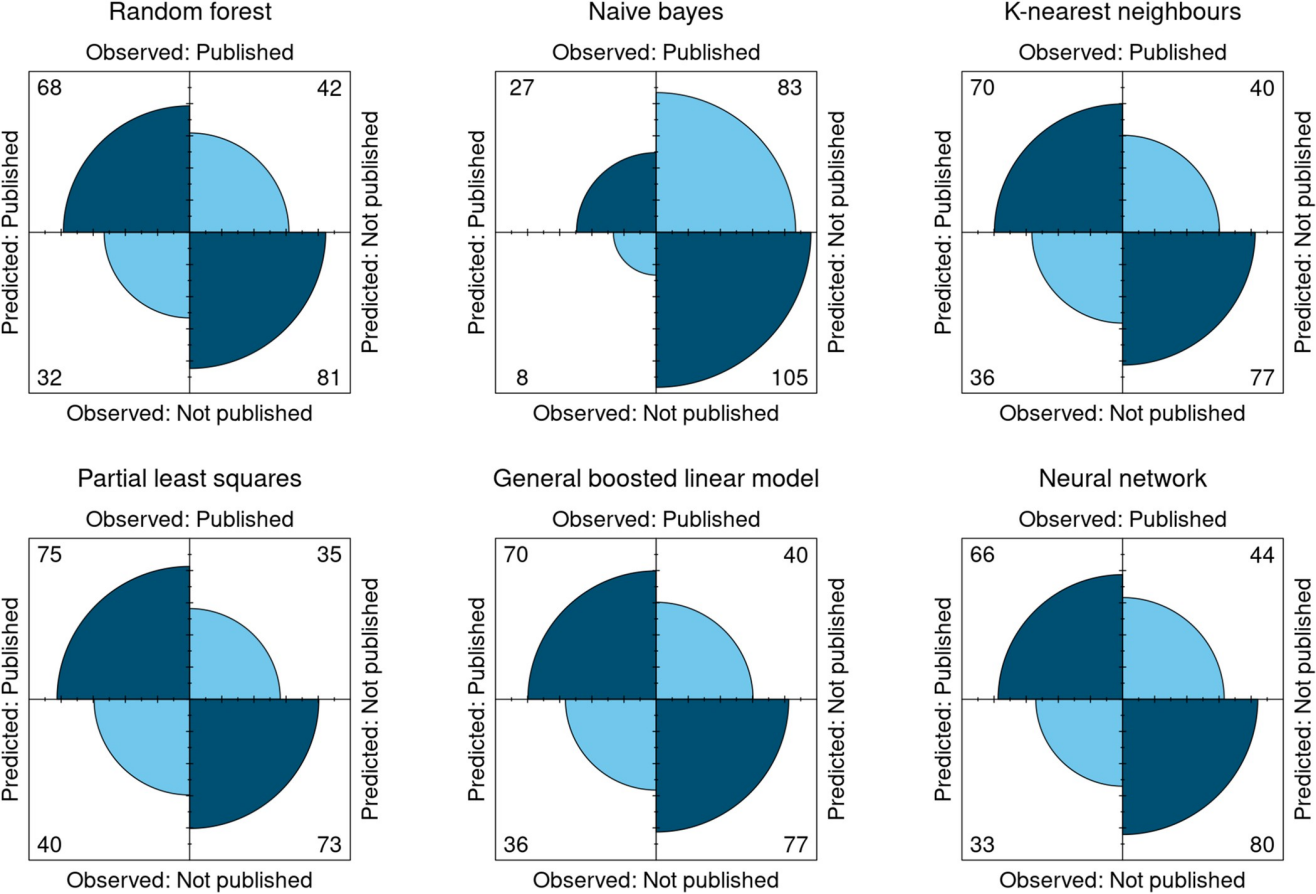
Confusion matrices of various algorithms applied to the testing data. Matrices to be interpreted like a 2-by-2 epidemiologic table, with true positives and true negatives on the top left and bottom right corners and false positives and false negatives in the top right and bottom left corners.

Fig 1 displays a network plot of the correlation between all features used to train the models. Fig 2 displays the ROC curves for all models during training and testing. Fig 3 displays the confusion matrices for all the models tested.

#### 3. 2. 1 Model results

During training, the models that generated the highest AUC metrics were the random forest and partial least squares models, with an AUC of 0.77 (SD 0.05) (Table 3). The worst model was the K-nearest neighbours, with an AUC of 0.75 (SD 0.05) and accuracy of 71.3% (SD 2.8). The partial least squares model had the highest accuracy during training of 75.5% (SD 4.7). All models had good AUC scores [20], with all scores being above 0.75. All models had similar sensitivity and specificity except the naive bayes model, with its sensitivity (correctly identifying published abstracts) being well below the others at 0.28 (SD 0.08) and specificity (correctly identifying unpublished abstracts) nearly perfect at 0.94 (SD 0.07).

**Table 3:** The mean of the resampled accuracy, area under the receiver operator curve (AUC), sensitivity, specificity, positive predictive value (PPN) and negative predictive value (NPV) during model training, cross validation, and testing.

Model	Accuracy (%)	AUC	Sensitivity	Specificity	PPV	NPV
Model Training and Cross Validation*						
Random forest	73.7 (5.62)	0.77 (0.05)	0.57 (0.08)	0.71 (0.07)	0.64 (0.07)	0.64 (0.05)
Naive bayes	71.3 (2.82)	0.76 (0.04)	0.28 (0.08)	0.94 (0.07)	0.68 (0.26)	0.61 (0.02)
K-nearest neighbours	73.1 (5.43)	0.75 (0.05)	0.63 (0.08)	0.63 (0.07)	0.62 (0.05)	0.64 (0.06)
Partial least squares	75.5 (4.67)	0.77 (0.05)	0.64 (0.07)	0.66 (0.08)	0.65 (0.05)	0.66 (0.05)
General boosted linear model	74.2 (4.71)	0.76 (0.05)	0.59 (0.06)	0.70 (0.07)	0.64 (0.06)	0.64 (0.04)
Neural network	74.1 (5.02)	0.76 (0.05)	0.63 (0.08)	0.65 (0.07)	0.63 (0.05)	0.65 (0.05)
Model Testing						
Random forest	67.0	0.69	0.62	0.72	0.68	0.66
Naive bayes	64.3	0.69	0.25	0.93	0.77	0.56
K-nearest neighbours	66.1	0.67	0.64	0.68	0.66	0.66
Partial least squares	66.5	0.69	0.68	0.65	0.65	0.68
General boosted linear model	66.1	0.69	0.64	0.68	0.66	0.66
Neural network	66.6	0.68	0.60	0.71	0.67	0.65

*Brackets during training and cross-validation indicate the standard deviation (SD) of each metric.

AUC: Area under the receiver operator curve, NA: Not applicable, NPV: Negative predictive value, PPV: Positive predictive value.

During testing, the models that generated the highest AUC metrics were the random forest, naive bayes, partial least squares, and general boosted linear models AUC of 0.69 (Table 3). The worst MLM was the K-nearest neighbours, with an AUC of 0.67 and accuracy of 66.1%. The random forest model had the highest accuracy of 67.0%. All models had acceptable AUC scores, with AUCs above 0.67 (Table 3). Like training, all models had similar sensitivity and specificity except the naive bayes model, with its sensitivity being much lesser than the others at 0.25 and specificity much higher at 0.93 (Table 3).

ANOVA analysis comparing the AUC metrics of different models during training and testing revealed a significant difference between all models (*p* < .0001). Post-hoc sampling demonstrated the largest difference between the random forest and naive bayes models, with a difference of -0.113 (95% confidence interval (CI): -0.115, -0.111), *p* < .0001.

Confusion matrices in Fig 3 provide detailed insight into the efficacy of the models on the test set. Each of these plots may be interpreted like a 2-by-2 epidemiologic table, with true positives (predicting publication when published) and true negatives (predicting not published when not published) on the top left and bottom right corners and false positives (predicting publication when not published) and false negatives (predicting no publication when published) in the top right and bottom left corners [21]. All models seem to perform similarly on the test set, with the random forest performing best and the naive bayes performing the worst. Despite the Naïve Bayes model having the second highest AUC, its distribution of predictive accuracy is distinct from the others (Fig 3).

#### 3. 2. 2 Feature importance

**Fig 4:**
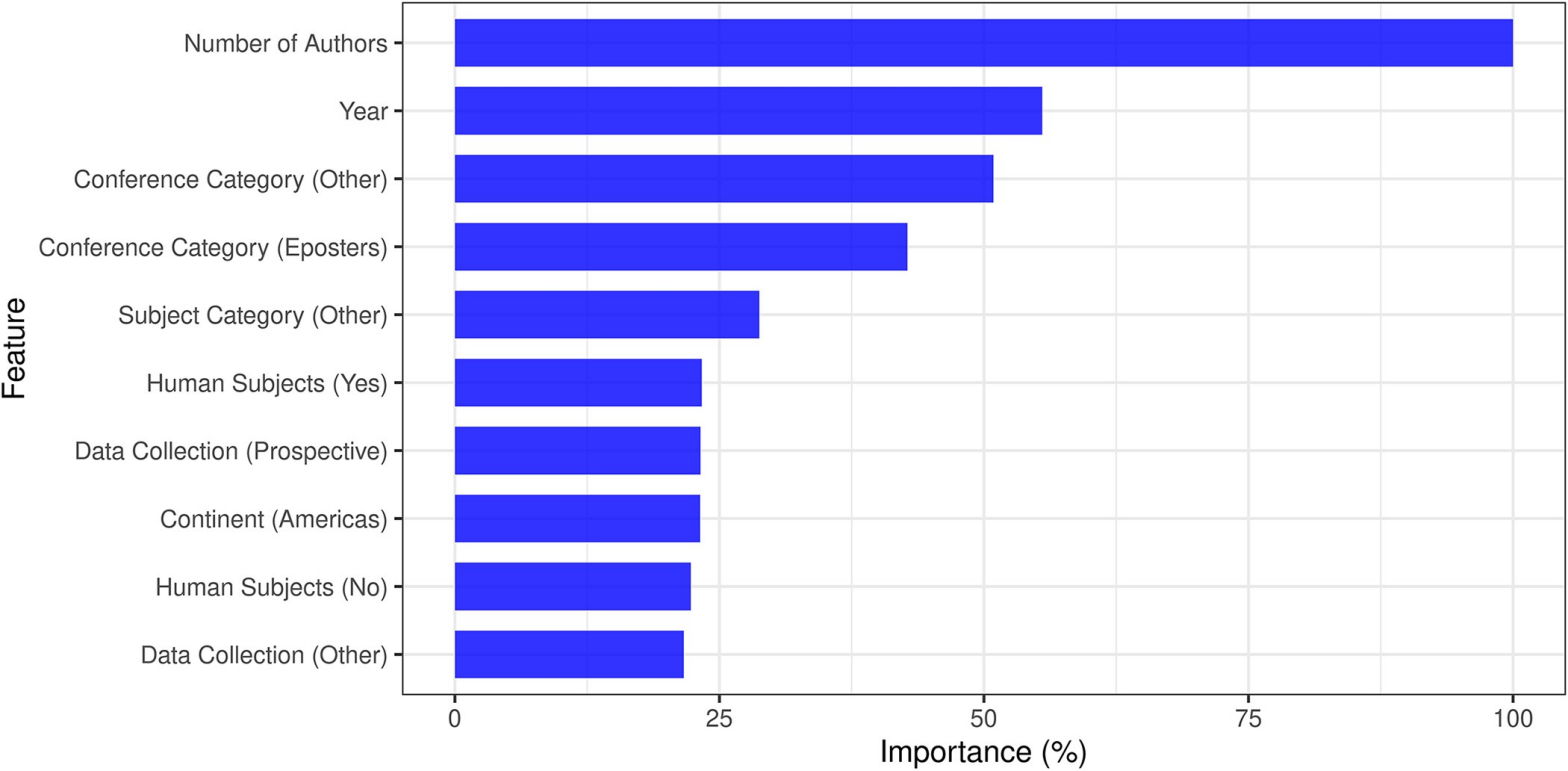
Bar plot representing the top ten most important features used by the random forest model. Importance is represented by percentage (%) normalized with respect to the most important feature.

The top ten features of the random forest model are displayed in Fig 4. According to the model, the features considered most important in descending order were number of authors (increased number increasing chance of publication), year, conference category, subject category, human subjects research, continent and data collection methodology.

## 4 Discussion

Our results show that approximately half of the abstracts at the NASS AGM from 2013–2015 were eventually published (~50% 5-year abstract-to-publication ratio). Our results are comparable to the 43.8% and 51% NASS AGM abstract publication rate reported by previously published studies [22,23].

To our knowledge, this was the first study using machine learning approaches to predict the publication of complete articles after abstract presentation at an academic meeting/conference. Our purpose was to show the feasibility of using ML to predict publication, which has been shown with the independent variables chosen. This should provide researchers a framework on which to build ML models that can answer future questions using other variables as deemed relevant for a particular question.

In this study, we tested different machine learning algorithms (random forest, partial least squares, k-nearest neighbours, naive bayes, neural network, general boosted linear model) to determine which of them performs best at predicting publication outcomes. The performance of each algorithm is measured by the metric AUC, with AUC values ranging between 0 and 1. The higher the AUC, the better the model is at predicting abstracts as published versus not published. For reference, an algorithm making random guesses as to which abstracts eventually get published will have an AUC of 0.5. With an AUC ranging from 0.69–0.77 (testing-training), the random forest machine learning algorithm performed 38%-54% better than random guessing. In addition, with accuracy ranging from 67.0%-73.7% (testing-training), the random forest model performed 37%-50.4% better than random guessing (49% accuracy). Despite not being near perfect in terms of AUC and accuracy (AUC >0.9, accuracy >90%), this demonstrates MLMs may generate predictions significantly better than random guessing. Given this is a relatively unexplored field, this improvement in performance is a noteworthy finding.

The top performing MLM (i.e., random forest) placed emphasis on the number of authors, year, conference category and subject category when generating the probability of prediction. A notable finding is that with regards to number of authors, abstracts with more authors were more likely to be published. This may be related to more authors indicating a higher degree of collaboration and thus more motivation for publication, but this is just conjecture. In addition, this insight could provide valuable information to future conference organizers when analysing the quality of their conferences. In addition, it could also help conference organizers improve the quality of research presented via stratifying the abstracts according to probability of publication, ensuring that research of potential greater scientific impact is highlighted as part of the conference’s program [24].

A common concern in machine learning literature is the quality and size of the dataset used. In this work, a detailed dataset was used, with 1119 abstracts and numerous features. In the previous studies of NASS abstracts [22,23], the type of presentation (poster vs. podium) and “Best” or “Outstanding” abstract designation were identified as factors that increased the likelihood of publication, which was different from the features deemed important by our study (Fig 4). These predictors of publication, nevertheless, were analysed using linear regression models. As the scientific community continues to incorporate machine learning into various aspects of research, methods of analysing conference abstracts should evolve as well. Machine learning enables us to deviate from the surface level analysis of publication rates/associations and attempt a detailed examination to uncover features that are important and useful for prediction, given its ability to model complex non-linear relationships between various features [22,25,26]. Our study, for example, used machine learning to identify specific predictive features, combining them to create a well performing predictive model, something which cannot be achieved with such granularity using standard statistical methods. Our study may provide some insight into some biases that may exist in acceptance of manuscripts for publication. For example, a major predictor of publication in the top performing model (random forest) was number of authors, which may speak to a biasing of more authors on abstracts increasing their likelihood of publication. This could provide insight into journals adjusting their requirements for acceptance by using these MLMs to create more equitable acceptance criteria. Ultimately, the application of machine learning to this field is an exciting opportunity for continuous improvement, development, and evaluation initiatives of scientific conferences.

This study has some limitations. Firstly, some features included in the algorithm (such as conference categories) vary widely from conference to conference. This means that these models would have limited utility where these features do not exist. However, future work should involve the use of adaptive MLMs utilizing neural networks, which would enable models to adapt according to the specifics of the dataset [27]. Secondly, the level of training of abstract authors was not captured. It is possible that authors earlier on in their academic/medical training would be more encouraged to publish work, indicating that this feature may be of important predictive value. In addition, it is well recognized that several other variables which are cognitively or results based as assessed by the reviewers are factors in final disposition of an abstract, such as the quality of the work or relevance to the conference theme [25,26]. These undoubtedly are significant variables which are not included in our study. Thirdly, despite the large dataset (1119 abstracts), the time frame (2013–2015) is not long enough to see general trends of publication over time, as demonstrated by the difference in publication rates of previous studies looking at NASS AGM abstracts (43.8% between 2010–2012 [22] and 51% between 2009–2011 [23]). This approach would be challenging to apply to some high impact scientific meetings that are smaller in size by virtue for meeting focus and target audience. The selection process of abstracts for presentation and specialized focus of NASS on interdisciplinary spine advances may differ to the mandate of other scientific medical conferences and the results of this study may not be generalizable to what may be the most important factors predicting publication for other societies. Additional information that may not be available based upon abstracts reviewed such as grant funding and other support may further improve the overall publication prediction. Fourthly, manuscript publication is not necessarily the goal for every abstract presented at research meetings. Many presentations are done to fulfil research requirements of residency or fellowship, which would hamper the ability of MLMs to predict subsequent publication. In addition, submissions to journals varies by topic. As such, certain topics may receive more submissions than others, resulting in acceptance and rejections rates. Furthermore, our study is unable to factor in how the laborious process of preparing a manuscript for publication factors in publication rates. However, the results of this study may provide insight into what manuscripts would be more successful in publication, perhaps streamlining this process. Lastly, deep learning frameworks were not used as these generally require dataset sizes on the order of tens of thousands. In the future, it would be interesting to gather this data over a larger interval e.g., 20 years to evaluate the effect of time on results and models, also allowing one to incorporate deep learning prediction models. Deep learning will allow one to model complex, nonlinear relationships which may arise with time better than more conventional ML models [28].

In conclusion, this study demonstrated that machine learning can be used to predict article publication from abstract presentations at the NASS AGMs. Cognitive variables that are based on the abstract content undoubtedly have significance on publication, which were not included in this study. However, having shown the feasibility of our models as is, further study using these cognitive based variables is indicated. The best model on the test set was the random forest algorithm with the highest AUC and accuracy. Future work should also incorporate a larger database over a longer period and include more features specific to the authors (such as level of training). Overall, incorporation of these MLMs can provide insight into the current biases that exist in publication, improve the quality of scientific meetings, and guide authors into creating the optimal manuscript suitable for publication.

## Supporting information

S1 Table Demographic breakdown of presented and published abstracts across the NASS AGM 2013–2015 for the training set. Total sample size was 896 abstracts.
(DOCX)

S2 Table Demographic breakdown of presented and published abstracts across the NASS AGM 2013–2015 for the testing set. Total sample size was 223 abstracts.
(DOCX)

S3 Table Hyperparameters of final Caret models. Parameters listed below are ones which were optimized. The other parameters were the default ones provided by the Caret package.
(DOCX)
