# Supplementary material for: Machine learning models can predict subsequent publication of North American Spine Society (NASS) annual general meeting abstracts
Source: PLoS One. 2023 Aug 22;18(8):e0289931. doi: 10.1371/journal.pone.0289931 (PMC10443859; doi:10.1371/journal.pone.0289931)
Supplement: S1 Table — Total sample size was 896 abstracts. (DOCX) [file pone.0289931.s001.docx]

**S1 Table.** **Demographic breakdown of presented and published abstracts across the NASS AGM 2013-2015 for the training set.** Total sample size was 896 abstracts.

| Variable | Presented (N) | Published (N) | Published (%) |  | Variable | Presented (N) | Published (N) | Published (%) |
| --- | --- | --- | --- | --- | --- | --- | --- | --- |
| Country of publication | | | |  | Study type | | | |
| USA | 688 | 344 | 50.00 |  | Other | 235 | 112 | 47.66 |
| Canada | 50 | 25 | 50.00 |  | Animal study | 22 | 10 | 45.45 |
| China | 39 | 20 | 51.28 |  | Basic science study | 61 | 33 | 54.10 |
| South Korea | 17 | 10 | 58.82 |  | Case-control | 28 | 16 | 57.14 |
| UK | 14 | 5 | 35.71 |  | Case-report/ series | 177 | 86 | 48.59 |
| Japan | 14 | 8 | 57.14 |  | Clinical review | 51 | 19 | 37.25 |
| Others | 74 | 30 | 40.54 |  | Cohort | 199 | 105 | 52.76 |
| Subject category | | | |  | Cross-sectional | 26 | 12 | 46.15 |
| Other | 411 | 213 | 51.82 |  | Randomized controlled trial | 68 | 34 | 50.00 |
| MIS | 110 | 50 | 45.45 |  | Systematic review | 29 | 15 | 51.72 |
| Degenerative | 100 | 54 | 54.00 |  | Data collection methodology | | | |
| Implant | 100 | 43 | 43.00 |  | Retrospective | 375 | 182 | 48.53 |
| Basic science | 89 | 41 | 46.07 |  | Prospective | 339 | 162 | 47.79 |
| Paediatrics | 41 | 19 | 46.34 |  | Other | 182 | 98 | 53.85 |
| Trauma | 45 | 22 | 48.89 |  | FDA approved indication | | | |
| Human subjects research | | | |  | Yes | 69 | 32 | 46.38 |
| Yes | 438 | 201 | 45.89 |  | No | 57 | 25 | 43.86 |
| No | 458 | 241 | 52.62 |  | n/a | 770 | 385 | 50.00 |

AGM: annual general meeting, FDA: food and drug administration, MIS: minimally invasive surgery, NASS: North American Spine Society.
