# Supplementary material for: Machine learning models can predict subsequent publication of North American Spine Society (NASS) annual general meeting abstracts
Source: PLoS One. 2023 Aug 22;18(8):e0289931. doi: 10.1371/journal.pone.0289931 (PMC10443859; doi:10.1371/journal.pone.0289931)
Supplement: S2 Table — Total sample size was 223 abstracts. (DOCX) [file pone.0289931.s002.docx]

**S2 Table. Demographic breakdown of presented and published abstracts across the NASS AGM 2013-2015 for the testing set.** Total sample size was 223 abstracts.

| Variable | Presented (N) | Published (N) | Published (%) |  | Variable | Presented (N) | Published (N) | Published (%) |
| --- | --- | --- | --- | --- | --- | --- | --- | --- |
| Country of publication | | | |  | Study type | | | |
| USA | 175 | 88 | 50.29 |  | Other | 63 | 32 | 50.79 |
| Canada | 8 | 5 | 62.50 |  | Animal study | 6 | 5 | 83.33 |
| China | 11 | 5 | 45.45 |  | Basic science study | 12 | 6 | 50.00 |
| South Korea | 7 | 3 | 42.86 |  | Case-control | 12 | 5 | 41.67 |
| UK | 6 | 3 | 50.00 |  | Case-report/ series | 37 | 17 | 45.95 |
| Japan | 3 | 1 | 33.33 |  | Clinical review | 11 | 5 | 45.45 |
| Others | 13 | 5 | 38.46 |  | Cohort | 46 | 25 | 54.35 |
| Subject category | | | |  | Cross-sectional | 8 | 3 | 37.50 |
| Other | 101 | 54 | 53.47 |  | Randomized controlled trial | 20 | 6 | 30.00 |
| MIS | 34 | 15 | 44.12 |  | Systematic review | 8 | 6 | 75.00 |
| Degenerative | 31 | 14 | 45.16 |  | Data collection methodology | | | |
| Implant | 21 | 9 | 42.86 |  | Retrospective | 96 | 46 | 47.92 |
| Basic science | 19 | 9 | 47.37 |  | Prospective | 86 | 41 | 47.67 |
| Paediatrics | 12 | 5 | 41.67 |  | Other | 41 | 23 | 56.10 |
| Trauma | 5 | 4 | 80.00 |  | FDA approved indication | | | |
| Human subjects research | | | |  | Yes | 25 | 12 | 50.00 |
| Yes | 110 | 48 | 43.63 |  | No | 12 | 6 | 50.00 |
| No | 113 | 62 | 54.87 |  | n/a | 186 | 92 | 49.46 |

AGM: annual general meeting, FDA: food and drug administration, MIS: minimally invasive surgery, NASS: North American Spine Society.
