# Supplementary material for: Machine learning models can predict subsequent publication of North American Spine Society (NASS) annual general meeting abstracts
Source: PLoS One. 2023 Aug 22;18(8):e0289931. doi: 10.1371/journal.pone.0289931 (PMC10443859; doi:10.1371/journal.pone.0289931)
Supplement: S3 Table — Parameters listed below are ones which were optimized. The other parameters were the default ones provided by the Caret package. (DOCX) [file pone.0289931.s003.docx]

**S3 Table. Hyperparameters of final Caret models**. Parameters listed below are ones which were optimized. The other parameters were the default ones provided by the Caret package.

| **Model** | **Parameters** |
| --- | --- |
| Random forest | mtry = 2, ntree = 500, nodesize = 1 |
| Naive bayes | laplace = 0, adjust = 1, usekernal = true |
| K-nearest neighbours | k = 9 |
| Partial least squares | ncomp = 1 |
| General boosted linear model | Shrinkage = 0.1, n. minobsinnode = 10, n.trees = 100, interaction.depth = 1 |
| Neural network | size = 5, decay = 0.1 |
